# Characterization of NCR1+ cells residing in lymphoid tissues in the gut of lambs indicates that the majority are NK cells

**DOI:** 10.1186/1297-9716-44-109

**Published:** 2013-11-13

**Authors:** Line Olsen, Preben Boysen, Caroline Piercey Åkesson, Gjermund Gunnes, Timothy Connelley, Anne K Storset, Arild Espenes

**Affiliations:** 1 Department of Basic Sciences and Aquatic Medicine, Norwegian School of Veterinary Science, Oslo, Norway; 2 Department of Food Safety and Infection Biology, Norwegian School of Veterinary Science, Oslo, Norway; 3 The Roslin Institute, Royal (Dick) School of Veterinary Studies, University of Edinburgh, Edinburgh, UK

## Abstract

Natural killer (NK) cells are important for immune protection of the gut mucosa. Previous studies have shown that under pathologic conditions NK cells, T cells and dendritic cells are found co-localised in secondary lymphoid organs where their interaction coordinates immune responses. However, in the gut-associated lymphoid tissues (GALTs), there are few detailed reports on the distribution of NK cells. Sheep harbour several types of organised lymphoid tissues in the gut that have different functions. The ileal Peyer’s patch (IPP) functions as a primary lymphoid tissue for B cell generation, while the jejunal Peyer’s patches (JPPs) and colon patches (CPs) are considered secondary lymphoid tissues. In the present study, we analysed tissues from healthy lambs by flow cytometry and *in situ* multicolour immunofluorescence, using recently described NCR1 antibodies to identify ovine NK cells. Most NCR1+ cells isolated from all tissues were negative for the pan T cell marker CD3, and thus comply with the general definition of NK cells. The majority of NCR1+ cells in blood as well as secondary lymphoid organs expressed CD16, but in the GALT around half of the NCR1+ cells were negative for CD16. A semi-quantitative morphometric study on tissue sections was used to compare the density of NK cells in four compartments of the IPPs, JPP and CPs. NCR1+ cells were found in all gut segments. Statistical analysis revealed significant differences between compartments of the primary lymphoid organ IPP and the secondary lymphoid organs of the JPPs and CP. NK cells co-localised and made close contact with T cells, dendritic cells and other NK cells, but did not show signs of proliferation. We conclude that NK cells are present in all investigated segments of the sheep gut, but that presence of other innate lymphoid cells expressing NCR1 cannot be excluded.

## Introduction

Natural killer (NK) cells are lymphocytes of the innate immune system traditionally known for their immediate cytotoxic activity against stressed, transformed or infected cells [1]. More recently, they have been shown to be present in lymphoid tissues, mucosal tissues and several other organ systems, where they exhibit direct effector functions as well as immunoregulatory actions on other cells through cytokine production [2-5]. By direct interactions with macrophages [6,7] or dendritic cells (DCs) [8], NK cells provide an early source of interferon-γ (IFNγ), which is necessary for T_H_1 polarization in the lymph nodes [9]. NK cells are known to be present in the intestinal mucosa of humans and mice, but their precise tissue compartmentalization and function have been a matter of debate, as other distinct lymphoid cell populations also express NK cell markers [10,11]. The distribution and phenotype of NK cells in the gut-associated lymphoid tissues (GALTs) of sheep have not yet been described.

The gut mucosa is constantly challenged with dietary and other exogenous antigens, and the immune system needs to react appropriately to both harmless and dangerous antigens. The organised lymphoid tissue of small intestinal Peyer’s patches (PPs) and the lymphoid patches of the colon (CPs), as well as the solitary lymphoid follicles present along the gastrointestinal tract, are the main inductive sites of the gut immune system. The lamina propria, which is found subepithelially throughout the gut, is regarded mainly as an effector site [12]. In lambs and calves, the continuous ileal PP (IPP) is responsible for the generation of B cells, and is considered a primary lymphoid tissue, unlike the jejunal PPs (JPPs) and CPs, which are recognised as secondary lymphoid tissues [13-16]. The PPs and CPs of sheep can be divided into immunologically relevant tissue compartments based on morphology, cellular composition, and function [13,17,18]. Each B cell containing follicle in the submucosa is surrounded by a capsule except on the luminal side where the follicle extends into the mucosa and blends with the dome. The dome contains myeloid and lymphoid cells and is covered by a specialized follicle-associated epithelium (FAE). Between the follicles and beneath the lamina muscularis mucosae is an area rich in T cells; the interfollicular area (IFA). A further compartment is the lamina propria, which is found along the whole length of the gut, and is present both within and beyond the borders of PPs. T- and B cells predominate in the IFA and follicle, respectively, of the sheep PPs [19-21]. In the lamb and sheep gut, DCs are mostly found in the dome, IFA and lamina propria and have been shown to express CD11c, CD205, and MHCII [22].

CD16+/CD14- lymphocytes in the blood of sheep have been identified as NK cells [23], and NK cells were later found to be more precisely defined by the expression of NCR1 (CD335, NKp46), a natural cytotoxicity receptor (NCR) [24]. The NCR1+/CD3- phenotype of lymphocytes has proven to be a reliable definition of NK cells in many species [25], but a flow cytometric technique has not been available for antibodies that label CD3 in sheep. In mice and humans, NK cells in tissues display aberrant phenotypes compared to NK cells in blood [2]. The presence of NK cells in lymphoid and mucosal tissues of sheep has not been previously described in detail.

The aim of this study was to characterize NCR1+ cells present in the intestine with respect to CD3 (pan-T cell marker) and CD16 (Fcγ receptor IIIa), and to compare the relative number of NCR1+ cells in different lymphoid tissues, with focus on GALT. The localisation of NCR1+ cells in the various lymphoid tissues of healthy sheep was investigated using multicolour immunofluorescence. Morphometry was further used to compare the density of NCR1+ cells in different compartments of JPPs, IPP and CPs. Finally, we wanted to investigate the spatial relation of NCR1+ cells to T cells and DCs.

## Materials and methods

### Animals and collection of tissues

Five (*n* = 5) clinically healthy young lambs, aged 31–43 days, were used in the present study. Three lambs belonged to the Norwegian white sheep breed, while 2 lambs were a cross between the Norwegian white sheep and the Texel breed. A second cohort was collected from an additional 5 older lambs at an abattoir. These animals were approximately 6 months of age and tissues were collected to perform multi-parameter flow cytometric labelling with an antibody against the cytoplasmic region of the human CD3 epsilon chain (cytCD3), reported to be cross-reactive to multiple species by the manufacturer. All tissues were collected immediately after the lambs were euthanized in a way that complied with the Norwegian Animal Welfare Act of 28 December 2009. An overview of the tissues and method of analysis are shown for each of the two age groups in Additional file 1.

### Antibodies

Primary antibodies used in the present study are shown in Table 1.

**Table 1:** Primary antibodies used in this study

**Antibody**	**Clone**	**Isotype**	**Specificity**	**Cellular expression**	**Source**	**Used in**	**Cross reactivity**^**a**^	**References**
Mouse anti-bovine NCR1	AKS4	IgG1	NCR1	Natural killer cells		Flow	Sheep, goat	[26-28]
	AKS6	IgG2b						
Rat anti-human CD3: Pacific blue (MCA1477)	CD3-12	IgG1	CD3ϵ	All T cells	AbD Serotec, Ltd., Oxford, UK	Flow	Cattle, pig, horse, dog, cat and other mammals	
Mouse anti-human CD16	KD1	IgG2a	CD16	NK cells, macrophages, some T cells		Flow	Sheep, cattle	[5,23]
Mouse anti-ovine NCR1	EC1.1	IgG1	NCR1	Natural killer cells		IF		[24]
Rabbit anti-human CD3 (A0452)		Poly-clonal	CD3ϵ	All T cells	Dako, Glostrup, Denmark	IF	Sheep, goat, cattle and others	[29]
Mouse anti-bovine CD205 (MCA1651)	CC98	IgG2b	CD205	Dendritic cells, some T cells, some B cells, some epithelial cells	AbD Serotec, Ltd., Oxford, UK	IF	Sheep	
Mouse anti-bovine CD11c (BAQ153A)	BAQ153A	IgM	CD11c	Dendritic cells, some macrophages	VMRD, Inc., WA, USA	IF	Sheep, goat, cattle and others	
Rabbit anti-Ki67 (ab15580)		Poly-clonal	Ki67	Proliferating cells	Abcam, Cambridge, UK	IF	Cattle, horse, dog, mouse, rat and other mammals	

^a^For antibodies not raised against sheep antigens.

IF, *in situ* immunofluorescence; Flow, flow cytometry.

### Flow cytometry

Peripheral blood mononuclear cells (MNC) were isolated using Lymphoprep (Axis-Shield, Dundee, UK) gradient, as previously described [26]. Tissues were prepared as previously described [30]; briefly, lymph nodes were sectioned through the cortex and medulla, PPs and CPs had the mucosal layer gently scraped off, and representative pieces of spleen and tonsils were sliced. These samples were disaggregated in a Medimachine (BD Biosciences, NJ, USA) and filtered. In tissues from the 5 older lambs collected from the abattoir, cells were incubated with LIVE/DEAD Fixable Far Red Dead Cell Stain Kit (Life Technologies, Ltd., Paisley, UK), according to the manufacturer’s instructions. Cells were surface stained with primary monoclonal antibodies (mAbs) against NCR1, then incubated briefly with normal goat serum for Fc receptor blocking, followed by PE-conjugated goat anti-mouse secondary antibodies (Southern Biotechnologies, AL, USA). For labeling of cytCD3, and following surface labeling, cells were permeabilized using Cytofix/Cytoperm kit (BD Biosciences), according to the manufacturer’s instructions, and finally incubated with an anti-CD3 antibody conjugated with Pacific blue. Labeled cells were resuspended in FACS Lysing Solution (BD Biosciences) and analyzed in a FACS Calibur (BD Biosciences) for the analysis done in 2010, or in a Gallios (Beckman Coulter, Inc., CA, USA) flow cytometer acquired in 2012. Data were processed using Kaluza 1.2 software (Beckman Coulter, Inc.).

### *In situ* immunofluorescence

The intestinal tissue samples for cryostat sectioning were placed with their mucosal side onto thin slices of liver to protect the mucosa against mechanical damage and to facilitate cryostat sectioning. All tissues were snap frozen in chlorodifluoromethane (Isceon^TM^) chilled with liquid nitrogen. The tissues were stored at −70 °C until further preparation. 7 μm thick cryosections were mounted onto poly-lysine-coated slides and stored at −70 °C before use. The sections were air dried at room temperature for one hour, fixed in acetone for ten minutes and then air dried for another ten minutes. The sections were rinsed and rehydrated before they were blocked for non-specific binding with 10% normal goat or sheep serum in phosphate-buffered saline (PBS)/0.5% Tween®80 (Sigma-Aldrich, MO, USA) for 30 min at room temperature. The blocking solution was carefully tapped off and a mixture composed of either two or three primary antibodies was applied and left at 4 °C overnight. Following a washing step, slides were incubated with isotype-specific Alexa Fluor® secondary antibodies (Molecular Probes, Inc., OR, USA) for one hour at room temperature. After a final washing, slides were mounted in polyvinyl alcohol at pH 8. All incubations were performed in a slowly rotating humid incubation chamber, and washing between each step was done in PBS/0.5% Tween®80 for five minutes. The primary and the secondary antibodies were diluted in the blocking solution. Control sections included (a) replacement of both primary and secondary antibodies by blocking solution, (b) replacement of primary antibodies by blocking solution and (c) replacement of primary antibody with an irrelevant antibody of the same isotype as the primary antibody.

### Microscopy

For quantitative analysis, sections labelled by immunofluorescence were stored in darkness at 4 °C until examination with a Leica DM RXA fluorescence microscope (Leica Microsystems, Wetzlar, Germany), and images were captured using a SPOT RT Slider^TM^ camera (Diagnostic Instruments, MI, USA) with SPOT 5.0 Advanced Software (Diagnostic Instruments). The filter cubes (Leica Microsystems, Wetzlar, Germany) A4 with excitation BP 360/40, L5 with excitation BP 480/40 and TX2 with excitation BP 560/40 were used for Alexa Fluor® 350, Alexa Fluor® 488 and Alexa Fluor® 594, respectively. In addition, images were captured using a Zeiss Axiovert 100 inverted microscope, equipped with an LSM 510 laser confocal unit with the Zeiss ZEN 2009 Software, Release Version 5.5 SP1 (Carl Zeiss, Jena, Germany). A combination of filters corresponding to the secondary antibodies Alexa Fluor® 488, Alexa Fluor® 546 and Alexa Fluor® 633 were used when capturing the confocal images.

### Evaluation of the immuno labelled slides

**Figure 1:**
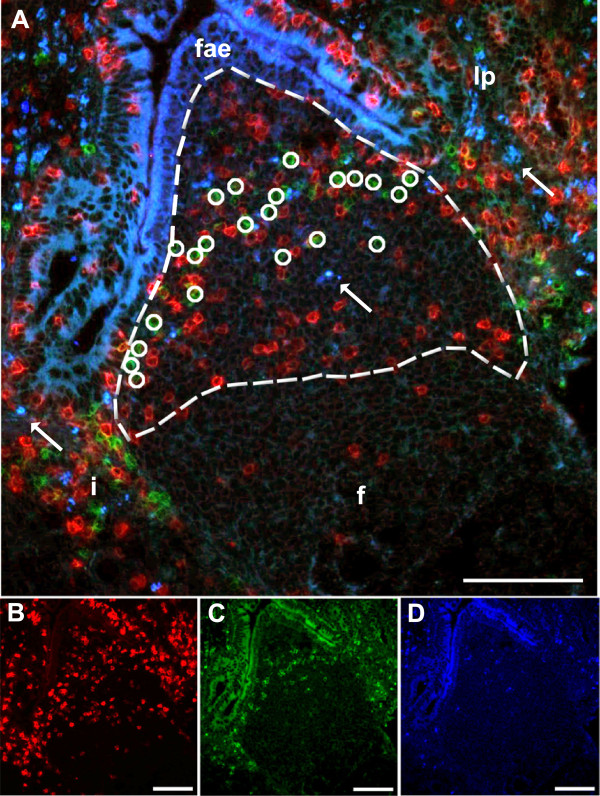
**Morphometric quantification of NCR1+ cells of the compartments in JPPs, IPP and CPs.** A dome of jejunal Peyer’s patch (delineated) is shown to illustrate the method of quantification. The area of interest was interactively defined based on tissue morphology; here shown with a merged image **(A)** of CD3 in red **(B)**, NCR1 in green **(C)** and unlabeled filter in blue **(D)**. The cells were counted manually (circles) and were distinct from the autofluorescence (arrows) particularly present in the lamina propria and dome. Follicle **(f)**, follicle-associated epithelium **(fae)**, interfollicular area **(i)**, lamina propria **(lp)**. Bars 100 μm.

To compare the density of NK cells in the different compartments of JPPs, IPP and CPs, images were obtained at original magnification of 200× from at least 4 individual areas for each compartment of each of the gut segments. The compartments studied were lymphoid follicles, IFA, domes and lamina propria of comparable pixel areas. The semi-quantative analysis was performed in Image-Pro® Plus software version 5.1 (Media Cybernetics, Inc., MD, USA) on JPEG files with a resolution of 1520 × 1080 pixels. An Image-Pro® Plus macro was written to reduce workload and minimise user bias. The macro loaded specified images, added pseudocolours and allowed the user to interactively define the area of interest and perform manual cell counts (Figure 1). Due to autofluorescence in the gut lamina propria, NCR1+ cells were identified based on the morphology of the circular membrane bound signal on medium sized cells and further by revealing the unwanted autofluorescent signals by merging at least two images taken with different colour filters. This method facilitated the differentiation between the autofluorescence (Figure 1, arrows) and the specific fluorescence from the secondary antibodies (Figure 1, circles).

### Statistical analysis

The flow cytometric data were assessed by nonparametric Wilcoxon rank-sum test for each pair, using the JMP 10.0 statistical software (SAS Institute, NC, USA). For the morphometric analysis of the density of NK cells, it was necessary to compensate for natural variability between individuals; therefore the non-parametric Wilcoxon-van Elteren [31] test was used to calculate the significance of differences between gut segments. Two-tailed tests were performed and differences were considered significant for *p*-values < 0.05.

## Results

### Quantification and characterization of NCR1+ cells by flow cytometry

**Figure 2:**
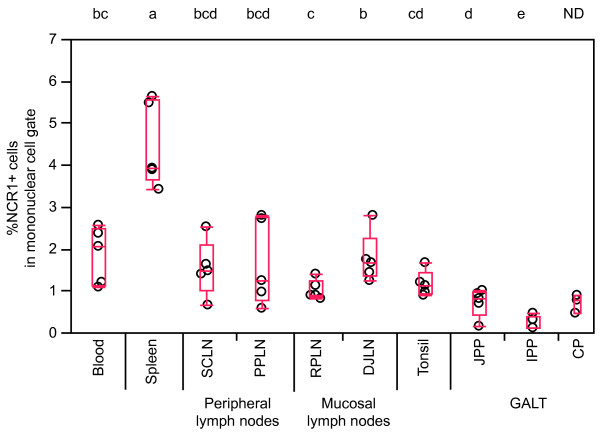
**Relative NCR1+ cell numbers in tissues of young lambs.** Mononuclear cells from peripheral blood or disaggregated tissues were analysed in flow cytometry. NCR1+ cells are stated as % of cells in the mononuclear gate. Dots represent individual animals (*n* = 5 and for colon patch *n* = 3), outlier boxes indicate median and quartiles, and whiskers indicate data points within 1.5× (interquartile range). Letters shared between groups indicate no significant difference according to the Wilcoxon Each Pair non-parametric rank sum test (*P* < 0.05). Not determined **(ND)**; superficial cervical lymph node **(SCLN)**; popliteal lymph node **(PPLN)**; retropharyngeal lymph node **(RPLN)**; distal jejunal lymph node **(DJLN)**; jejunal Peyer’s patch **(JPP)**; ileal Peyer’s patch **(IPP)**; colon patch **(CP)**; gut-associated lymphoid tissue **(GALT)**.

**Figure 3:**
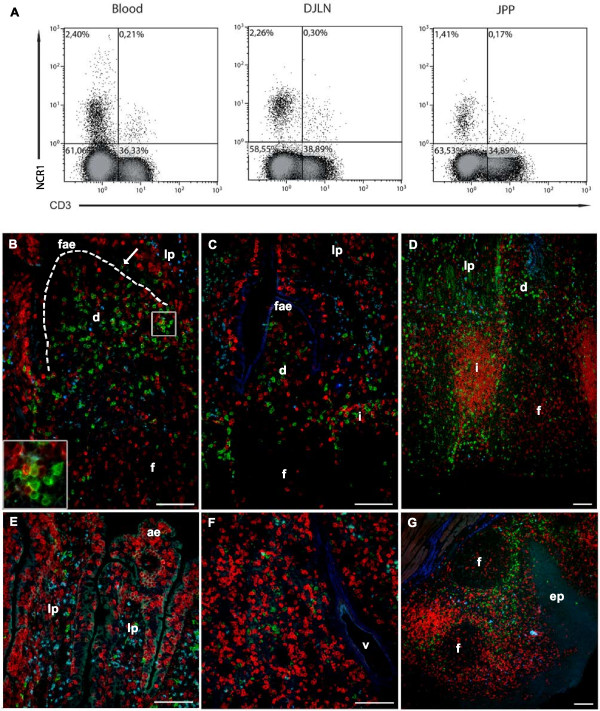
**NCR1+ cells in relation to CD3. (A)** Flow cytometric plots of viable mononuclear cells from blood, distal jejunal lymph node **(DJLN)** and jejunal Peyer’s patch **(JPP)** of lambs. Cells were labeled with fixable Live/Dead (aqua) dye, followed by labeling for NCR1, then permeabilization and labeling for a conserved intracellular CD3 epitope (CytCD3). Plots are representative of five animals, at approx. 6 months of age. **(B-G)** Representative figures of 5 one month old lambs, showing the *in situ* distribution of NCR1+/CD3- cells and T cells in jejunal Peyer’s patch **(B)**, ileal Peyer’s patch **(C)**, colon patch **(D)**, jejunum **(E)**, superficial cervical lymph node **(F)** and tonsil **(G)**. A two-colour fluorescent labeling with the NK cell antibody NCR1 (green) and the pan T cell marker CD3 (red) was used. The NCR1+/CD3- cells were present at a moderate to large amount in the compartments of dome **(d)**, interfollicular area **(i)**, at a moderate amount in lamina propria **(lp)**, while very few NCR1+ cells were found in the follicle **(f)**. In the follicle-associated epithelium **(fae)**, NCR1+/CD3- cells were observed intermittently (arrow). NCR1+/CD3+ cells were not observed, but NK cells and T cells were localised in the same areas, and were often seen in close contact with each other (inset; merged membranes appear yellow). To illustrate autofluorescence, photos were taken with a blue filter which did not detect signals from the secondary antibodies used in the study. The basal lamina in dome is delineated **(B)**. Absorptive epithelium **(ae)**; epithelium **(ep)**; vessel **(v)**. Bars 50 μm **(B-G)**.

The quantity of NCR1+ cells was given as the number of positive cells in relation to the total number of MNC gated in a FSC/SSC scatter plot. Peripheral blood contained 1-3% NCR1+ cells and the spleen 3-6% (Figure 2). Lymph nodes, draining mucosal or peripheral tissues, as well as tonsils and CPs, contained NCR1+ cells at proportions comparable to blood, mostly around 1-2%. The gut PPs and particularly IPP contained less than 1% NCR1+ cells, which was markedly lower than blood. To assess whether NCR1 could be present on a subclass of T cells, we labelled cells isolated from various tissues collected from 5 older abattoir lambs using an antibody against the cytCD3. This was done because mAbs against extracellular regions of ovine CD3 were not available. This additional analysis showed that almost all NCR1+ cells separated from these tissues was cytCD3- (Figure 3A). A minimal population of NCR1+ cells expressing CD3 (< 1% of MNC) was observed in JPP (0.4% of MNC), distal jejunal lymph node and blood (both 0.2% of MNC), but not in retropharyngeal lymph node (< 0.05% of MNC) (Figure 3A and not shown); however, such double expression was not observed by *in situ* immunofluorescence analysis (presented below and in Figure 3B-G).

**Figure 4:**
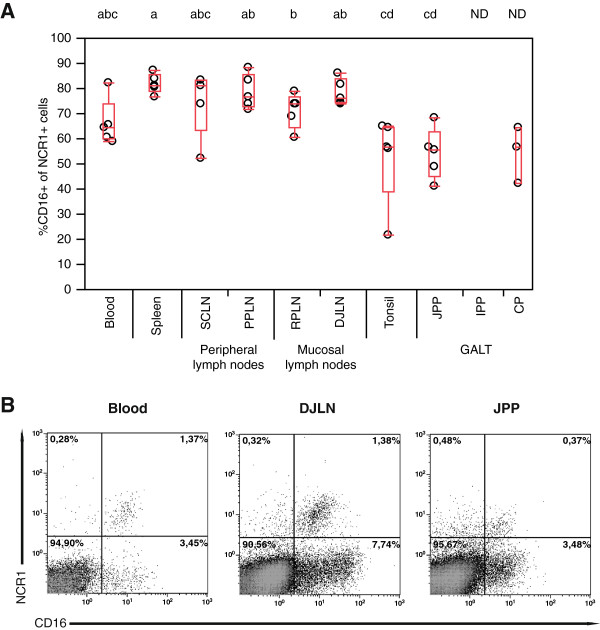
**CD16 expression on NCR1+ cells. (A)** Proportions of NCR1+ cells that express CD16. Plot elements and statistics as in Figure 2. Not determined **(ND)**. **(B)** Corresponding flow cytometric plots, showing CD16 in relation to NCR1 expression in mononuclear cells, representing one out of five lambs. Not determined **(ND)**; superficial cervical lymph node **(SCLN)**; popliteal lymph node **(PPLN)**; retropharyngeal lymph node **(RPLN)**; distal jejunal lymph node **(DJLN)**; jejunal Peyer’s patch **(JPP)**; ileal Peyer’s patch **(IPP)**; colon patch **(CP)**; gut-associated lymphoid tissue **(GALT)**.

The literature reports considerable variability in the presence of CD16 on NK cells between organs and between species, so we went on to measure the expression of this marker. The great majority of NCR1+ cells in blood, spleen and lymph nodes expressed CD16, whereas in tonsils, JPPs and CPs only approximately half of the NCR1+ cells were CD16+ (Figure 4). The low number of NCR1+ cells obtained from IPP made subset characterization unreliable.

### *In situ* study of NCR1+ cells in GALT, tonsils and lymph nodes by immunofluorescence

NCR1+ cells were present in all organs studied, and these cells generally did not co-label with antibodies against CD3 (Figure 3B-G). NCR1+/CD3- cells were present in all investigated segments of the gut. The lamina propria contained moderate numbers of NCR1+/CD3- cells that were scattered throughout the stroma. A low number of these cells was located within the absorptive epithelium, but never observed in the lacteals. In the IFA, numerous NCR1+/CD3- cells were evenly distributed. Sometimes these cells were found within the fibres of the lamina muscularis mucosae, but none was observed in the submucosal lymphatics. A high number of NCR1+/CD3- cells were evenly distributed in the dome and a few cells were occasionally observed within the FAE (Figure 3B, arrow). A few NCR1+/CD3- cells were found in the centre of the follicles in JPPs and CPs, while the follicles of IPP were chiefly devoid of this cell population. In the lymph nodes and tonsils, NCR1+/CD3- cells were present in abundance in the diffuse extra-follicular areas, particularly in regions close to the follicles, while few or none were found in the follicles (Figure 3F-G). In the lymph nodes, the density of NCR1+/CD3- cells gradually decreased from the cortex towards the medulla (Figure 3F). Attempts to analyse ovine tissues with the CD16 mAb were unsuccessful by the *in situ* immunofluorescent technique used as no NCR1+ cells did co-label with the CD16 mAb.

### Morphometric and statistical analysis of the density of NCR1+/CD3- cells in compartments of JPPs, IPP and CPs

**Figure 5:**
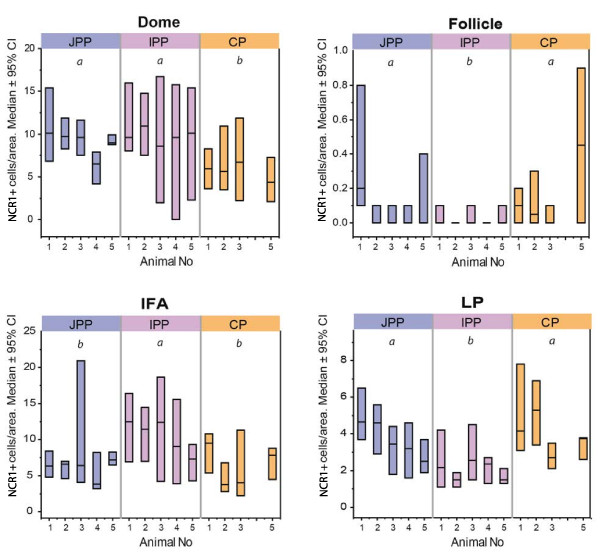
**Statistical analysis of the density of NCR1+ cells in JPPs, IPP and CPs.** The results are expressed as median with 95% confidence interval constructed using the Bernoulli-Wilcoxon procedure. Note that animal nr. 4 has missing data for colon patch due to the macroscopic inability to localise it in this animal. Within each compartment, letters shared between gut segments indicate no significant difference (*P* < 0.05). Jejunal Peyer’s patch **(JPP)**; ileal Peyer’s patch **(IPP)**; colon patch **(CP)**; interfollicular area **(IFA)**; lamina propria **(LP)**.

Statistical analysis of the morphometric observations in GALT revealed significant differences between gut segments with respect to the density of NCR1+/CD3- cells in the four compartments (Figure 5). The density of NCR1+/CD3- cells in IFA of IPP was higher compared with this tissue compartment in JPPs and CPs, while the lamina propria and follicles had a lower density in IPP compared with these tissue compartments in JPPs and CPs. The NCR1+/CD3- cell density of the dome in IPP and JPPs was similar, and the domes in both these gut segments had a higher density than the domes of CPs.

### Localisation of NCR1+/CD3- cells in relation to T cells and DCs

**Figure 6:**
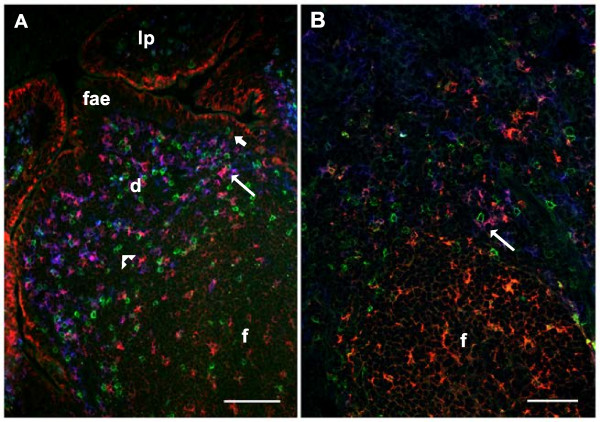
**NCR1+ cells in relation to DCs.***In situ* co-localisation studies of NCR1+ cells and dendritic cells, represented here in the jejunal Peyer’s patch **(A)** and superficial cervical lymph node **(B)**. NCR1+ (green) cells were found in the same compartments as CD205+ (red)/CD11c+ (blue) dendritic cells (pink-purple) and were sometimes observed in close contact with each other (long arrow). Note that occasionally, the NK cells were found intraepithelially, including in the follicle-associated epithelium **(fae)** (short arrow) and were often observed to be in cell-to-cell contact with each other (arrowhead). Lamina propria **(lp)**; dome **(d)**; follicle **(f)**. Bars 100 μm.

Generally, NCR1+/CD3- cells were found in areas where T cells were abundant (Figure 3B-G). However, the number of NCR1+/CD3- cells did not always seem to correspond to the number of T cells as some areas had a relatively high number of T cells and a low number of NCR1+/CD3- cells, and vice versa. In all lymphoid tissues, including GALT, lymph nodes and tonsils, the NCR1+/CD3- cells had a similar tissue compartment distribution as the CD11c+/CD205+ DCs (Figure 6). Often, distinct cell-to-cell contact between NCR1+/CD3- cells and T cells (Figure 3B, inset) or DCs (Figure 6, long arrow) was observed. In addition, there were many cell-to-cell contacts between the NCR1+/CD3- cells (Figures 3B-G and Figure 6, arrowhead). To study whether this cell contact was associated with cell division, a double immunofluorescent labelling with NCR1 and Ki67 was performed. No NCR1+ cells, including those in close contact, were found to co-label with Ki67, indicating that NK cells are not undergoing cell division in these tissues (not shown).

## Discussion

The NCR molecule NCR1 is a conserved cell membrane marker identifying NK cells in many mammalian species [25,26,32,33]. The present study utilized immunofluorescence with combinations of antibodies against NCR1 and other relevant immune cell markers, to characterize ovine NK cells, their *in situ* distribution and relation to other immune cells in various lymphoid tissues with focus on GALT. Basic information on a possible cellular overlap between NCR1 and the pan-T cell marker CD3 was obtained by flow cytometry and *in situ* immunofluorescence. As found in human intestine and in lymphoid tissues of mice [25,32], only a minor population of the ovine NCR1+ cells co-labelled for CD3. This finding was consistent in blood and all lymphoid tissues including GALT and it is the first time NCR1+ cells in ovine tissues have been shown to be CD3 negative. Thus, the present study shows that NCR1+ cells are mainly NK cells and not a subpopulation of T cells, which is a finding that adds support to previous studies characterizing ovine NCR1+ cells as typical NK cells [24].

CD16 is an activation receptor present on classical NK cells mediating recognition of antibody-opsonized targets. Flow cytometric analysis of NCR1+/CD3- cells showed that most NCR1+ cells in blood, spleen and lymph nodes were CD16+. This observation is in accordance with previous studies of NK cells in the blood of sheep [23,24], as well as in blood, lymph nodes and spleen of cattle and cats [5,33]. Compared with NK cells in blood, lymph nodes and spleen, the JPPs and CPs showed lower, but still relatively high expression of CD16. We were unable to support these findings *in situ* as the CD16 mAb was not compatible with the immunofluorescence technique used. In humans, a CD16+ NK cell subset dominates in blood, spleen and liver, while a CD16 low/- population dominates in lymph nodes, uterus, skin, and in the mucosal tissues such as tonsils and intestinal wall [2,3,34-37]. The latter “regulatory” NK cell population has strong IFNγ producing capabilities and has a poor cytotoxic potential, but the absence of the CD16 molecule does not seem to be constant and expression of CD16 can be up-regulated during inflammatory bowel disease in humans [38]. A previous report in sheep showed that cultured ovine NK cells expressed CD16 in the majority of cells but at a slightly lower level [24], in contrast to cattle where up-regulation was seen [5]. In mice, a CD16 antibody has only recently become available, resulting in conflicting reports whether or not CD16 is present on NK cells in mice at all [39,40]. Thus with respect to CD16 expression, plasticity and species differences are apparent, owing to genetic as well as environmental factors.

Recently, NK cells in the gut mucosa have been grouped under innate lymphoid cells (ILCs), a category of cells that plays a significant role in gut immunity as well as tissue development and remodelling [10]. The ILCs have been organized into three main groups named ILC1, ILC2 and ILC3, based on their cytokine producing profile [11]. Classical NK cells belong to the ILC1 group. ILC2s are NCR negative and important for helminth expulsion. ILC3s are important for lymphoid tissue organogenesis and the protective function of epithelial cells during infection [10]. The expression of NCRs has been proposed as a signature of most ILC3s across species. The ILC3 cells may be particularly important for immunity against pathogenic bacteria and may also play a role in inflammatory bowel disease [11]. In human intestine, the low expression of CD16 under non-inflammatory conditions appears to be common for all ILCs, including classical NK cells [35,37]. Although the definition of ILC groups in sheep is lacking, it is likely that the NCR1+/CD16+ population represents classical (ILC1) cells, while NCR1+/CD16- cell population in sheep could be associated with any of the ILC groups. Unfortunately, a scarcity of antibodies against ILC markers (including RORγt, CD117, CD127 and IL-22) hampers the detailed analysis of such cells in sheep.

**Figure 7:**
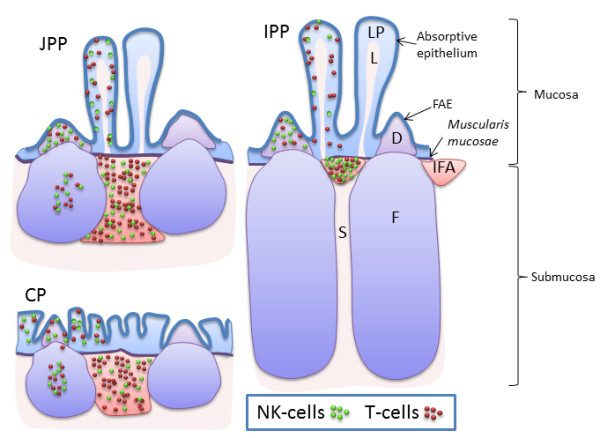
**Illustration of the density of NK cells in ovine JPPs, IPP and CP.** In the B cell containing follicles located in the submucosa, a higher density was found in jejunal Peyer’s patches **(JPPs)** and colon patch **(CP)** compared to the almost NK cell empty follicles of the ileal Peyer’s patch **(IPP)**. In the T cells rich area between the follicles and beneath the lamina muscularis mucosae; the interfollicular area (IFA), the density of NK cells was higher in IPP than in JPPs and CP, while in lamina propria of the villi had a lower density in IPP compared to JPPs and CP. In the dome area, covering the follicles, the density was lower in CP compared to IPP and JPPs. Dome **(D)**; follicle **(F)**; follicle-associated epithelium **(FAE)**; interfollicular area **(IFA)**; lacteal in villus **(L)**; lamina propria **(LP)**; submucosa **(S)**.

The relative numbers of NK cells quantified by flow cytometry in blood, lymph nodes and spleen of sheep were largely in accordance with previous studies [23,24]. Mucosal and non-mucosal lymph nodes appeared to have similar levels of NK cells, unlike observations in cattle, where a higher level was found in the non-mucosal lymph nodes [5]. A distinct population of NK cells was observed by flow cytometry in JPPs, CPs and tonsils. In IPP, a very low proportion of mononuclear cells were found to be NCR1+, although in tissue sections a substantial number of such cells was detected. This discrepancy can probably be explained by the structure of this organ, as when applying flow cytometry, the vast number of B cells of the follicles will exert a major dilution effect on the NK cells (Figure 7).

A general description of the presence of NK cells in the ovine gastrointestinal tract has previously been published [24]. However, the different segments of GALT in sheep do not only differ in structure and cell composition, but also in function and development [13,17,41,42]. Therefore, a more detailed investigation of the distribution and density of NK cells were undertaken and these results were correlated to the immune function of the different GALT segments. The lymphoid follicles of the JPPs and CPs had a higher density of NK cells than IPP. The JPPs and CPs are secondary lymphoid tissues [16,17,43-46], and it is plausible that NK cells in the follicles may provide priming signals to T_H_1 CD4+ T cells found here [15,41,42,47,48], as reported in lymph nodes [9]. In contrast, the lower density of NK cells in the follicles of IPP could be related to the function as a primary lymphoid organ where the follicles are generation sites for circulating B lymphocytes before involution at puberty [16]. Similarly, the lamina propria of the ovine IPP was shown to have less density of NK cells compared to the JPPs and CPs, and this could indicate that the lamina propria of IPP is a less efficient effector site compared with lamina propria of the secondary lymphoid tissues. The finding could be consistent with the presence of reduced local cellular and humoral immune responses in IPP [49], and possibly lowered innate immune function such as reduced NK cell density found in the present study. The relatively high, but variable density of NCR1+ cells in the dome and IFA of IPP, JPPs and CPs could, in addition to immune responses, also be related to organ development, as previously reported for ILC3s in thymus development [50]. Thus, further sub-typing of NCR1+ cells in different GALT segments and compartments in these organs is needed to distinguish conventional NK cells from other ILCs in sheep.

Similar to the co-localisation of NK cells, DCs and T cells in lymphoid organs of humans and mice [4,25,51], we observed DCs and NK cells in close proximity in the T cell rich areas, like the dome, lamina propria and IFA of the gut, in addition to the diffuse extra-follicular areas of lymph nodes and tonsils. The reciprocal crosstalk between NK cells and DCs plays a pivotal role in tolerogenic regulation under normal conditions [52] and innate cytolytic immune responses against infections [53] and cancer [4,54,55]. Distinct NK cell immunological synapses have been described for the different lytic, inhibitory or regulatory functions of these cells [56,57]. We propose the possibility that the NCR1+/CD3- cells detected in this study execute a similar DC-NK cell communication demonstrated in mice and humans, as these two cell populations reside in the same compartments and were demonstrated to be in close contact, both in gut, lymph nodes and tonsils.

In conclusion we have found ovine NCR1+ cells to be chiefly CD3-/CD16+ in blood, spleen and lymph nodes, and to a lesser extent also in GALT organs. Accordingly, we postulate that most of these NCR1+ cells are classical NK cells of the ILC1 group. NK cells resided in compartments rich in, and in close contact to both T cells and DCs, consistent with their need for cell-to-cell contact for important functions in immunoregulatory activities. Differences found in the density of NK cells between the primary and secondary lymphoid tissues of the GALTs indicate dissimilar functions, and since about half of the NCR1+ cells in JPPs, CPs and tonsils were CD16 negative, it is necessary to determine if this cell phenotype will be associated with other ILC groups. Moreover, it remains to be elucidated whether all or some of the NK cells under steady state conditions in lambs are cytolytic, inhibitory or regulatory in function and whether these NK cells will change expression patterns upon infection.

## Abbreviations

NK: Natural killer; DC: Dendritic cell; IFNγ: Interferon gamma; GALT: Gut-associated lymphoid tissue; PP: Peyer’s patch; CP: Colon patch; IPP: Ileal Peyer’s patch; JPP: Jejunal Peyer’s patch; FAE: Follicle-associated epithelium; IFA: Interfollicular area; NCR: Natural cytotoxicity receptor; cytCD3: Cytoplasmic region of CD3; MNC: Mononuclear cell; mAb: Monoclonal antibody; PBS: Phosphate-buffered saline; ILC: Innate lymphoid cell.

## Competing interests

The authors declare they have no competing interests.

## Authors’ contributions

LO, PB, CPÅ, GG, AKS, and AE designed research, performed research, analyzed data, and contributed to the manuscript. LO wrote the manuscript. TC made contribution to conception and design, and helped to draft the manuscript. All authors read and approved the final manuscript.

## Supplementary Material

Additional file 1 **Tissues obtained and the method of analysis used in this study.** The table gives a list of all tissues obtained from the animals euthanized at the two time-points and their respective method of analysis. Click here for file
