# Supplementary material for: Characterization of NCR1+ cells residing in lymphoid tissues in the gut of lambs indicates that the majority are NK cells
Source: Vet Res. 2013 Nov 13;44(1):109. doi: 10.1186/1297-9716-44-109 (PMC4176090; doi:10.1186/1297-9716-44-109)
Supplement: Additional file 1 — Tissues obtained and the method of analysis used in this study. The table gives a list of all tissues obtained from the animals euthanized at the two time-points and their respective method of analysis. [file 1297-9716-44-109-S1.doc]

| **Year** | **Organ (Abbreviation)** | **Analysis^b^** |
| --- | --- | --- |
| 2010 | Blood | F |
|  | Spleen | F |
|  | Tonsil | IF |
|  | Jejunal PP (JPP) | F, IF |
|  | Ileal PP (IPP) | F, IF |
|  | Colonic PP^a^ (CP) | F, IF |
|  | Superficial cervical LN (SCLN) | F, IF |
|  | Popliteal LN (PPLN) | F, IF |
|  | Retropharyngeal LN (RPLN) | F |
|  | Distal jejunal LN (DJLN) | F, IF |
|  | Jejunal LN (JJLN) | IF |
| 2012 | Blood | F |
|  | Distal jejunal LN (DJLN) | F |
|  | Retropharyngeal LN (RPLN) | F |
|  | Jejunal PP (JJLN) | F |
| ^a^Missing from animal No.4.  ^b^Flow cytometry (F); In situ immunofluorescence (IF)  Peyer’s patch (PP); Lymph node (LN) | | |
